# Evaluation of efficiency and safety of combined loratadine and budesonide in patients with anaphylactic rhinitis: A protocol for systematic review and meta-analysis

**DOI:** 10.1097/MD.0000000000028851

**Published:** 2022-05-06

**Authors:** Jing Zhang, Dan Kan

**Affiliations:** Department of Otorhinolaryngology, WuHan Puren Hospital, Wuhan, Hubei, China.

**Keywords:** anaphylactic rhinitis, budesonide, efficiency, loratadine, meta

## Abstract

**Background:**

Among the most prevalent allergic conditions that affect children is anaphylactic rhinitis (AR). It is capable of leading to physical as well as mental health issues. Concomitant use of *loratadine* and *budesonide* may improve symptoms of AR more than treatment with either drug alone. To assess the efficacy and safety of combined *loratadine* and *budesonide* for patients experiencing AR is the aim of this study.

**Methods:**

We will apply 2 independent authors in six databases, including EMBASE, Pub Med, Web of Science, China National Knowledge Infrastructure, WanFang Database, Chinese Scientific Journal Database (VIP database). Studies evaluating the efficacy and safety of combined loratadine and budesonide in patients with AR will include studies published between inception and Dec 2021. Accordingly, the data will have to be in English and Chinese. For the selection of data extraction, the studies and risk of bias assessment will be completed by 2 independent authors. Accordingly, data synthesis will be conducted through RevMan 5.3 software. The study will establish heterogeneity using the *I*^2^ test. Without correct data or information, there is a need for Publication bias, which is assessed by performing the Begg and Egger test and generating a funnel plot.

**Results:**

The study provides a trustable clinical foundation for *loratadine* and *budesonide* for AR treatment.

**OSF registration number:** DOI 10.17605/OSF.IO/M2RFG

**Ethics and dissemination:** Because the present study is founded on existing studies, it does not require ethics approval.

## 1 Introduction

Anaphylactic rhinitis (AR) has a substantial global health issue. In essence, it is 1 of the most typical forms of non-infectious rhinitis, accounting for an estimated 10 to 30 percent of all adults as well as 40 percent of young persons. Past epidemiological studies have demonstrated that AR continues to increase globally. For example, the WHO has estimated that about 400 million people suffer from AR worldwide.^[1]^ Accordingly, AR is caused by a specific IgE-mediated allergic reaction, mainly in the nasal mucosa; hence, characterized by “stuffy, itchy, runny or runny nose, and sneezing.”^[2]^ Apart from specific allergen immunotherapy, currently available therapeutic approaches, such as antihistamines and corticosteroids, tend to focus on symptom relief, and although they do not provide a permanent solution, they remain first-line treatment.^[3]^

*Loratadine* refers to a medication with high permeability and low water solubility, and it is usually used to treat allergic symptoms.^[4–6]^ Essentially, by oxidized low-density lipoproteins, the drug is capable of reducing endothelial inflammation produced and has some level of protective impacts.^[7]^ Accordingly, *loratadine* can selectively inhibit H1 receptors situated primarily on respiratory smooth muscle cells and those that do not cross the blood-brain obstruction. At the same time, it is helpful in many situations for relieving allergic symptoms.^[8]^ Even though *loratadine* displays a clinical efficacy to treat AR, it is necessary to enhance its effect.^[9]^

*Budesonide* entails a nebulized glucocorticoid utilized in treating asthma and AR. It caused substantial decreasing adhesion molecules levels, including MIP-2 and ICAM-1, released from endothelial cells and wounded epithelial; thus, triggering neutrophil infiltration and macrophages.^[10,11]^ Accordingly, *budesonide* is capable of decreasing proinflammatory cytokines (IL-1β, TNF-α, IL-6), increasing the levels of IL-10; thus, antagonizing the impacts of earlier cytokines as well as reducing cell apoptosis.^[11–13]^ However, the safety and efficiency of combining *loratadine* and *budesonide* in patients with AR have not been systematically verified. To this end, this review aims to empirically assess the safety and efficacy of combining *loratadine* and *budesonide* to treat patients experiencing AR and build reliable evidence and valuable references for clinicians and researchers to make better medical decisions. This will help n conducting further studies on the topic.

## 2 Materials and methods

The study is registered with the Open Science Framework (OSF, accession number DOI 10.17605/OSF.IO/M2RFG). Systematic reviews and meta-analyses as required by the project statement are our preference.

### 2.1 Inclusion criteria

#### 2.1.1 Type of study

We only included randomized controlled trials published or registered before December 2021. However, we will exclude prospective randomized controlled trials, review articles, case reports and other studies that do not meet this requirement.

#### 2.1.2 Type of participants

We will include participants of different age ranges, participants of all types of AR, regardless of their nationality, gender, race, occupation, education, severity, or etiology.

#### 2.1.3 Type of interventions

We will give the therapy of *loratadine* combined with *budesonide* to the treatment group, whereas the control group will receive only *loratadine*, *budesonide*, placebo, or they will not receive any form of treatment. We will have no limit to dose, frequency, or treatment duration.

#### 2.1.4 Types of outcome measures

The clinical improvement of AR symptoms, rhinoconjunctivitis-related quality of life, occurrence of adversative events, and utilization of rescue medicine is our anticipated outcome.

### 2.2 Search methods for identification of studies

#### 2.2.1 Electronics searches

We will use 2 independent in six distinct databases: Web of Science, PubMed, EMBASE, WanFang Database, China National Knowledge Infrastructure, and Chinese Scientific Journal Database (VIP database). They will assess the evaluation of efficacy as well as safety of combined *loratadine* and *budesonide* in patients with AR, published from inception to December 2021. We will only regard articles published in English and Chinese. A search in PubMed was performed using the following terms “(‘loratadine∗’ OR ‘budesonide∗’) AND (‘rhinitis∗’ OR ‘allergic rhinitis’ OR ‘anaphylactic rhinitis’) AND (random∗ OR trial OR ‘randomized controlled trial’ OR ‘randomized controlled trial’.

#### 2.2.2 Searching other resources

The following clinical trial registries are what we will use to establish ongoing trials: The ClinicalTrials.gov, the Chinese clinical registry, and Google Scholarship. We will also retrieve helpful but incomplete information from the contact trial personnel.

### 2.3 Data extraction and management

We will use 2 independent authors to double-check and gather all the qualifying studies and transfer them into RevMan software. We will use a pre-defined data acquisition form to enter details, for instance “author, journal, treatment indication, population characteristics, total and per-arm sample size, publication year, comparator dose and omalizumab, study duration, and mode of administration.” In case of a disagreement between the authors, a third author will mediate to help reach a consensus.

### 2.4 Assessment of risk of bias

Two independent authors will be used to perform a systematic review of each of the studies for the bias risk. The authors will use the Cochrane Handbook. In particular, they will rely on 6 domains, including “reporting, the bias of selection, detection, performance, attrition and other sources.” They will rate trials for every field as “high risk, low risk or unclear after evaluation.” The authors involved in the study will also be contacted to clear any missing information. In case of a disagreement between the authors, a third author will mediate to help reach a consensus

### 2.5 Measures of treatment effect

We pooled the study-specific estimates by utilizing fixed and random-effects models and estimated the standardized summary mean differences and the relative risks, as well as the 95% confidence intervals correspondingly. We noticed that the standardized mean difference was the mean change per standard deviation, which is critical for comparing scores in various scales.

### 2.6 Dealing with missing data

We will get in touch with respective authors where there is missing information or incompleteness of data. We will wait for 1 month after sending an email to the original author to reply. After 1 month, we will exclude the incomplete data from the analysis if the author does not respond or provide the necessary data.

### 2.7 Heterogeneity assessment and subgroup analysis

We use *I*^2^ (ranging from 0% to 100%) to assess the presence and degree of heterogeneity. We used a subgroup analysis for further investigation where there is high heterogeneity. We intend to use the formal test for subgroup interactions in RevMan V.5.3.

### 2.8 Sensitivity analysis

A sensitivity analysis will be used to conduct the robustness of the critical decisions made during the monitoring review process.

### 2.9 Publication bias

We used a visual inspection of the Egger test and Begg funnel plots to evaluate all potential small study effects, such as an indication of publication bias.

## 3 Discussion

AR is 1 of the most common allergic diseases worldwide, affecting 10% to 40% of the global population.^[14]^ In particular, many people seem to experience the effects of the negative impacts of AR. Therefore, patients might resort to using medication to relieve these uncomfortable symptoms. Nevertheless, some of the most common adverse events with intranasal formulations were “poor taste, nasal burning, sedation, increased cost relative to oral formulations and more frequent dosing.” In most cases, doctors consider side effects based on the initiation of intranasal antihistamines. On this foundation, we need to strengthen our randomized controlled study on the safety and effectiveness of combining *loratadine* and *budesonide* to treat patients with AR in future studies. We also need to establish a significant scientific basis to apply this approach to clinical treatment. This will be crucial in the provision of a solid scientific foundation to use this approach in the clinical therapy of AR.

## Author contributions

**Conceptualization:** Jing Zhang, Dan Kan.

**Data curation:** Jing Zhang, Dan Kan.

**Formal analysis:** Jing Zhang, Dan Kan.

**Funding acquisition:** Jing Zhang, Dan Kan.

**Investigation:** Jing Zhang.

**Methodology:** Jing Zhang, Dan Kan.

**Project administration:** Jing Zhang.

**Resources:** Dan Kan.

**Software:** Jing Zhang, Dan Kan.

**Supervision:** Jing Zhang.

**Validation:** Jing Zhang, Dan Kan.

**Visualization:** Jing Zhang.

**Writing – original draft:** Jing Zhang, Dan Kan.

**Writing – review & editing:** Dan Kan.
